# Operating Range for a Combined, Building-Scale Liquid Air Energy Storage and Expansion System: Energy and Exergy Analysis

**DOI:** 10.3390/e20100770

**Published:** 2018-10-08

**Authors:** Todd A. Howe, Anthony G. Pollman, Anthony J. Gannon

**Affiliations:** Graduate School of Engineering and Applied Sciences, Naval Postgraduate School, Monterey, CA 93943, USA

**Keywords:** liquid air energy storage, energy analysis, exergy analysis, cryogenic system

## Abstract

This paper presents the results of an ideal theoretical energy and exergy analysis for a combined, building scale Liquid Air Energy Storage (LAES) and expansion turbine system. This work identifies the upper bounds of energy and exergy efficiency for the combined LAES-expansion system which has not been investigated. The system uses the simple Linde-Hampson and pre-cooled Linde-Hampson cycles for the liquefaction subsystem and direct expansion method, with and without heating above ambient temperature, for the energy production subsystem. In addition, the paper highlights the effectiveness of precooling air for liquefaction and heating air beyond ambient temperature for energy production. Finally, analysis of the system components is presented with an aim toward identifying components that have the greatest impact on energy and exergy efficiencies in an ideal environment. This work highlights the engineering trade-space and serves as a prescription for determining the merit or measures of effectiveness for an engineered LAES system in terms of energy and exergy. The analytical approach presented in this paper may be applied to other LAES configurations in order to identify optimal operating points in terms of energy and exergy efficiencies.

## 1. Introduction

Liquid air energy storage (LAES) is a developing thermal electrical energy storage technology and is a promising addition to other long-term storage technologies like pumped hydroelectric storage (PHS) and compressed air energy storage (CAES) [1,2]. LAES has a higher energy density than PHS and four to six times the energy density of CAES at 200 bar [1,3]. Studies have shown LAES is capable of higher round trip efficiency than CAES [4]. In addition, LAES has an advantage over CAES and PHS due to not being constrained to geographical features [5]. Although, micro-CAES systems are not constrained to geographic features and are effective in distributed power networks [6,7]. Highview Power Storage developed a 300 kW LAES pilot plant in Slough, Scotland and have a 10 MW commercial demonstration plant planned [8].

LAES has two main subsystems, the air liquefaction subsystem and energy production subsystem. In the liquefaction subsystem, air is compressed, cooled, and expanded which produces liquid air. Multiple configurations like Linde-Hampson cycle, Claude cycle, Heylandt cycle, Collins cycle, and more, liquefy and store air [9]. The energy production system has multiple configurations like direct expansion method, indirect Rankine cycle, indirect Brayton cycle, and other variations that may be implemented [10]. Storage is commonly included as a third subsystem, although this paper includes liquid air storage in the liquefaction subsystem.

There are multiple thermodynamic studies on liquid air energy storage available in literature. Early research in 1977 compressed air to 7 atm, dehumidified air and compressed further to 49 atm and cooled for storage; then repressurized liquid air to 80 atm before expanding through a turbine, which gave an efficiency up to 72% [11]. Guizzi et al. [12] conducted a thermodynamic analysis of a LAES system with a multi-stage compressor and turbine, and a storage subsystem, which resulted in a round-trip efficiency of 54–55%. A similar system and analysis conducted by [13] showed round trip efficiencies from 45–57% when adjusting the inlet pressure to a JT valve. Another analysis demonstrated the effects on LAES energy efficiency when adjusting the compressor efficiency, compressor discharge pressure, and cryogenic pump discharge pressure [14]. Additional work has focused on the liquefaction subsystem. Abdo et al. conducted a thermodynamic analysis on the Linde-Hampson cycle, Claude cycle, and Collins cycle and showed Claude and Collins cycles both having higher overall efficiencies than the Linde-Hampson cycle [15]. Yu et al. conducted an exergy analysis of a Linde-Hampson cycle with an ejector and showed the addition of the ejector reduced the total exergy destruction in the cycle [16].

This paper presents the results of an ideal theoretical energy and exergy analysis for a liquid air energy storage system. Energy is conserved through all processes and systems, but this is untrue for exergy [17]. Unlike exergy analysis, energy analysis does not account for the quality of energy; this makes exergy analysis useful when searching for areas of improvement within a system [18,19]. The quality of energy, or exergy, is the “maximum work which can be obtained from a given form of energy using the environmental parameters as the reference state” [20]. The importance of exergy analysis, opposed to only energy analysis, is analyzing the irreversibilities in the system and its components. The analysis in this paper explores the upper bounds of energy efficiency and exergetic efficiency of the combined LAES-expansion system, highlights the effectiveness of precooling for liquefaction, heating air beyond ambient temperature for expansion, and analysis of the system components.

## 2. System Description

**Figure 1:**
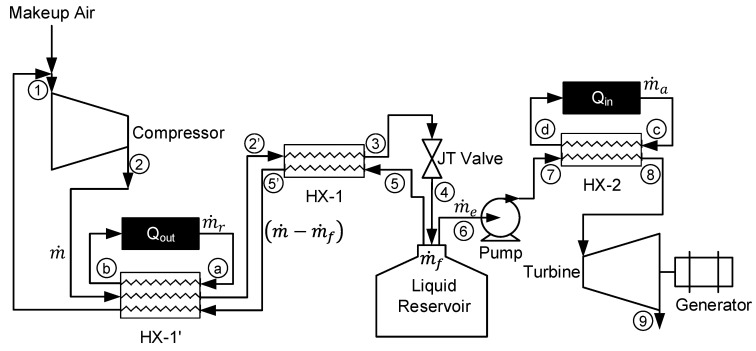
System diagram of a liquid air energy storage system.

The liquid air energy storage system analyzed in this paper investigates the two different liquefaction subsystems, the simple Linde-Hampson cycle and the pre-cooled Linde-Hampson cycle; and two different energy production subsystems, a direct expansion method and the direct expansion method with additional heat added. Figure 1 displays the complete LAES system with alternative options to use either type of liquefaction subsystem or energy production subsystem.

The simple Linde-Hampson cycle consists of a compressor, heat exchanger (HX-1), Joule-Thomson (JT) valve, and liquid reservoir. At steady state, mixing of makeup air and return air occurs prior to entering the compressor at state 1. Air is compressed to state 2 and then cooled in HX-1 to state 3. The cooled high-pressure air is expanded through the JT valve to state 4 where it is a 2-phase mixture. The liquid reservoir stores liquid air at state 6 for later use by the energy production subsystem and the gas returns to HX-1 at state 5 providing the cooling from states 2 to 3. The pre-cooled Linde-Hampson system adds an additional heat exchanger, HX-1′. The subsystem providing the additional cooling in HX-1′ is treated as a black box and only the required Q_out_ is calculated to achieve a desired state 2′ temperature.

The direct expansion method for the energy production subsystem consists of a cryogenic pump, heat exchanger (HX-2), and turbine with a generator. The cryogenic pump pumps liquid air from the liquid reservoir to a desired pressure to state 7. Heat exchanger 2 heats pressurized liquid air to ambient temperature by the surrounding heat or available waste heat to state 8. A turbine then expands the evaporated air to generate electricity to state 9. The direct expansion method is a simple but an inefficient method to extract liquid air [10]. The system can extract additional energy from the liquid air when heated beyond ambient temperature prior to entering the turbine. The Q_in_ represents the additional heat required to heat the liquid air above ambient pressure. Similar to HX-1′, this is treated as a black box where the subsystem to achieve the required Q_in_ to reach a desired state 8 temperature is not considered.

**Figure 2:**
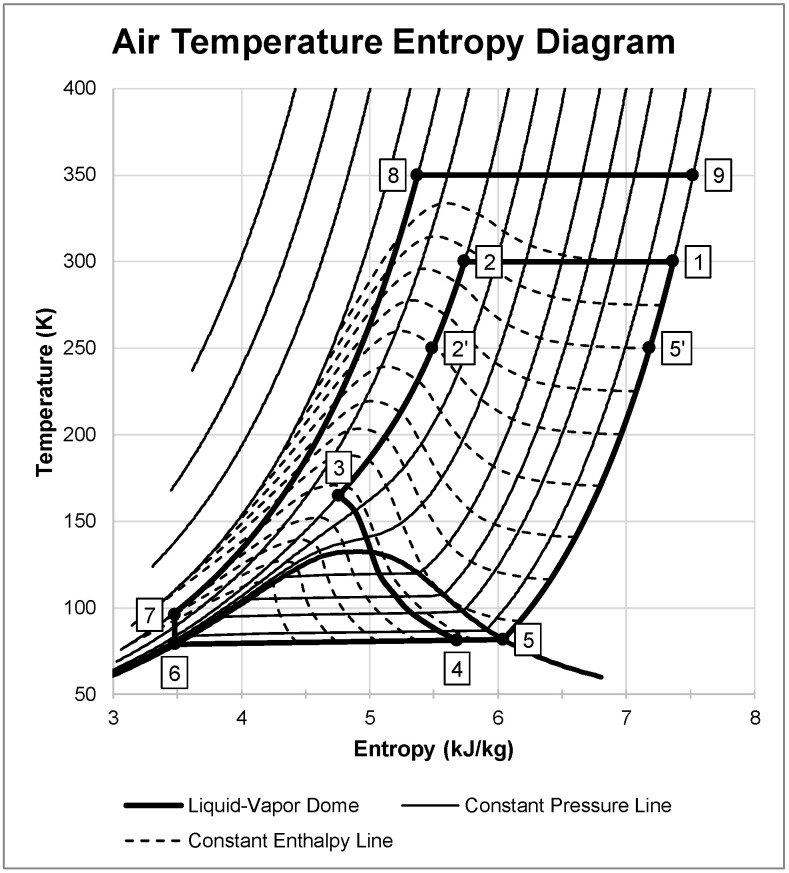
Air temperature entropy diagram showing each state in the LAES system.

Multiple combinations of state 2 pressures, state 2′ temperatures, state 7 pressures, and state 8 temperatures can be used in this LAES system. Figure 2 shows an example of the system dynamics of the LAES system with one possible combination. The figure shows the liquefaction subsystem as states 1 through 5′ where ambient air it pressurizes air to 20 MPa, precools to 250 K, and expands to generate approximately 21.3% liquid yield. The remaining gas passes through both heat exchangers, cooling the incoming air. States 6 through 9 represent the energy production subsystem. The subsystem pumps liquid air to 100 MPa, heats beyond ambient temperature to 350 K, and expands isothermally to state 9. This figure displays an ideal case for the LAES system where there is isothermal compression and expansion, 100% effective heat exchangers, and an isentropic cryogenic pump.

## 3. System Energy Analysis

As previously stated, this paper is investigating the upper bounds of an ideal LAES system. Therefore, all components are ideal components, the compressor isothermally compresses fluid, pumps isentropically compress fluids, heat exchangers are 100% effective, the turbine isothermally expands fluid, and there are no losses in lines. This section and the following section are organized by looking at each subsystem individually and then as a complete system.

### 3.1. Liquefaction Subsystem

The key information needed for the liquefaction subsystem is the required work for the compressor, the liquid yield, and required heat rejection from HX-1′. Using the first and second law on the compressor, the work per unit mass required to compress air from state 1 to 2 is [9,17,21]:

(1) $$\frac{{\dot{W}}_{c}}{\dot{m}}=T_{1}\left(s_{2}-s_{1}\right)+\left(h_{1}-h_{2}\right)$$

This analysis calculated the liquid yield for the simple Linde-Hampson subsystem using a control volume encompassing HX-1, the JT valve, and liquid reservoir. The control volume excludes HX-1′ and therefore, state 2 equals state 2′ and state 5′ equals state 1. Since there is no work or heat transferred to or from this control volume, the energy balance is [9,21]:

(2) $$\dot{m}h_{2}={\dot{m}}_{f}h_{f}+\left(\dot{m}-{\dot{m}}_{f}\right)h_{1}$$

The ratio of liquid mass flow rate to mass flow rate provides the liquid yield for the simple Linde-Hampson subsystem:

(3) $$Y=\frac{{\dot{m}}_{f}}{\dot{m}}=\frac{h_{2}-h_{1}}{h_{f}-h_{1}}$$

The compressor work per unit mass liquefied is therefore the compressor work per unit mass divided by the liquid yield:

(4) $$\frac{{\dot{W}}_{c}}{{\dot{m}}_{f}}=\frac{{\dot{W}}_{C}}{\dot{m}Y}=\left[T_{1}\left(s_{2}-s_{1}\right)+\left(h_{1}-h_{2}\right)\right]\left(\frac{h_{f}-h_{1}}{h_{2}-h_{1}}\right)$$

The pre-cooled Linde-Hampson system requires the use of a second heat exchanger (HX-1′) [9]. There are two cooling streams, ${\dot{Q}}_{\mathrm{out}}$ stream and the returning air stream. The required ${\dot{Q}}_{\mathrm{out}}$ for any desired temperature at state 2′ is based on the energy balance of HX-1′ [9]:

(5) $${\dot{m}}_{r}h_{a}+\dot{m}h_{2}+\left(\dot{m}-{\dot{m}}_{f}\right)h_{5^{\prime }}={\dot{m}}_{r}h_{b}+\dot{m}h_{2^{\prime }}+\left(\dot{m}-{\dot{m}}_{f}\right)h_{1}$$

Assuming the mass flow rate through the liquefaction subsystem, $\dot{m}$, is equal to the mass flow rate in the black box subsystem, ${\dot{m}}_{r}$, rearranging the equation and solving for Q_out_ per unit mass gives:

(6) $$\frac{{\dot{Q}}_{out}}{\dot{m}}=h_{a}-h_{b}=\left(h_{2^{\prime }}-h_{2}\right)+\left(1-Y\right)\left(h_{1}-h_{5^{\prime }}\right)$$

Using the previous control volume but now including HX-1′, the energy balance is [9]:

(7) $${\dot{m}}_{r}h_{a}+\dot{m}h_{2}={\dot{m}}_{r}h_{b}+\left(\dot{m}-{\dot{m}}_{f}\right)h_{1}+{\dot{m}}_{f}h_{6}$$

Defining the black box subsystem mass flow-rate ratio as [9]:

(8) $$r=\frac{{\dot{m}}_{r}}{\dot{m}}$$

The yield of the pre-cooled Linde-Hampson subsystem is therefore:

(9) $$Y=\frac{{\dot{m}}_{f}}{\dot{m}}=\frac{h_{2}-h_{1}}{h_{f}-h_{1}}+r\frac{h_{a}-h_{b}}{h_{f}-h_{1}}$$

As Equation (9) shows, pre-cooling provides additional liquid yield. This analysis uses the assumption that the ratio, *r*, is equal to one and 100% effective heat exchangers. Therefore, the liquid yield of the subsystem is unchanged from Equation (3).

### 3.2. Energy Production Subsystem

The energy calculations needed for the direct expansion method is the cryogenic pump work and the turbine work. The analysis assumes that the mass flow rate of the energy production subsystem is equal to the mass flow rate of the liquefaction subsystem. Additionally, the analysis assumes the pump isentropically compresses liquid air and there are no heat losses in the pump. Using these assumptions, the pump work is defined as:

(10) $$\frac{{\dot{W}}_{p}}{\dot{m}}=h_{6}-h_{7}$$

Assuming isothermal expansion, the turbine work is:

(11) $$\frac{{\dot{W}}_{t}}{\dot{m}}=T_{8}\left(s_{9}-s_{8}\right)+\left(h_{8}-h_{9}\right)$$

No work is associated with HX-2 for the direct expansion method. This ideal system assumes the compressed liquid air reaches ambient temperature through a heat exchanger by means of the surrounding air or by waste heat recovery. Although this is not practical in a real system, this is a step in ensuring the analysis determines the true upper bounds of the system. Providing additional heat to HX-2 allows state 8 to reach temperatures beyond ambient temperature. The analysis assumes additional heat required is only heat required beyond 300 K at a particular pressure. Therefore, assuming the mass flow rate of the heating source is equivalent to the mass flow rate of the energy production subsystem, the equation for additional heat is:

(12) $$\frac{{\dot{Q}}_{in}}{\dot{m}}=h_{8}-h_{300K}$$

where $h_{300K}$ is the enthalpy of air at a temperature of 300 K at a given pressure.

### 3.3. Complete LAES System

The first law measure of performance for the complete LAES system is the overall system efficiency, or round trip efficiency. The equation to calculate the overall system efficiency is dependent on the subsystems used. The simple Linde-Hampson subsystem with direct expansion method has work inputs to the compressor and pump from Equations (4) and (10), respectively. The heat rejection required from ${\dot{Q}}_{\mathrm{out}}$ in HX-1′ for the pre-cooling Linde-Hampson subsystem requires additional work to be performed. This work is calculated as the work required for a Carnot refrigerator [17]:

(13) $$\frac{{\dot{W}}_{HX_{1^{\prime }}}}{\dot{m_{f}}}=\frac{{\dot{Q}}_{out}}{{\dot{m}}_{f}}\left(\frac{T_{1}}{T_{5^{\prime }}}-1\right)$$

Given liquid air is the working fluid, using work per unit mass liquefied is preferred for energy efficiency calculations. When the temperature of state 8 exceeds ambient temperatures, additional work is required to add the necessary heat to achieve this temperature. This additional work is defined as the work required for a Carnot heat pump [17]:

(14) $$\frac{{\dot{W}}_{HX_{2}}}{{\dot{m}}_{e}}=\frac{{\dot{Q}}_{in}}{{\dot{m}}_{e}}\left(1-\frac{T_{7}}{T_{8}}\right)$$

The overall system efficiency is the ratio of work output to inputs:

(15) ηsys=W˙t(W˙CY)+W˙p+W˙HX1′+W˙HX2

## 4. System Exergy Analysis

The below exergy analysis uses a steady-state exergy rate balance to calculate the exergy destruction within a given component, as seen in Equation (16) [17]:

(16) $$0=\sum \left(1-\frac{T_{0}}{T_{j}}\right){\dot{Q}}_{j}-{\dot{W}}_{cv}+\sum {\dot{m}}_{i}\psi_{i}-\sum {\dot{m}}_{e}\psi_{e}-\dot{I}$$

where the exergy flow, ψ, is defined as [17]:

(17) $$\psi_{i}=h_{i}-h_{0}-T_{0}\left(s_{i}-s_{0}\right)+\frac{V_{i}^{2}}{2}+gz_{i}$$

This analysis assumes kinetic and potential energy terms are negligible, and therefore Equation (17) reduces to:

(18) $$\psi_{i}=h_{i}-h_{0}-T_{0}\left(s_{i}-s_{0}\right)$$

The reference state temperature, $T_{0}$, is assumed to be 300 K at a pressure of 0.101325 MPa. The analysis will present the calculations of the exergy destruction rate, $\dot{I}$, and exergetic efficiency, *ε*, for each component and the system.

### 4.1. Liquefaction Subsystem

From state 1 to 2, there is no temperature change, and therefore, the compressor exergy destruction rate is:

(19) I˙cm˙=−W˙cm˙+ψ1−ψ2

Substituting in Equation (18):

(20) I˙cm˙=−W˙cvm˙+h1−h2−T0(s1−s2)

The exergetic efficiency for the compressor is [17,22]:

(21) εc=ψ2−ψ1(−W˙cvm˙)

Assuming the mass flow rates $\dot{m}={\dot{m}}_{r}$ with no work or heat loss, the exergy destruction rate for HX-1′ is [23]:

(22) I˙HX1′m˙=(ψ2−ψ2′)+(ψa−ψb)+(1−Y)(ψ5′−ψ1)

The exergetic efficiency for HX-1′ is the ratio of the exergy increase of the hot stream to the exergy decrease in the cold streams [17,22]:

(23) εHX1′=(ψ2−ψ2′)(ψb−ψa)+(1−Y)(ψ1−ψ5′)

The exergy destruction and exergetic efficiency for HX-1 is:

(24) I˙HX1m˙=(ψ2′−ψ3)+(1−Y)(ψ5−ψ5′)

(25) εHX1=(ψ2′−ψ3)(1−Y)(ψ5′−ψ5)

The analysis assumes there is no work or heat transfer to the surroundings for fluid flow through the JT valve. In addition, there is no change in enthalpy during a throttling process; therefore, the exergy destruction rate reduces to:

(26) I˙JTm˙=T0(s4−s3)

The JT valve exergetic efficiency is defined as the ratio of the exergy flow out to the exergy flow in [22]:

(27) εJT=ψ4ψ3

The analysis completes the calculation for the simple Linde-Hampson subsystem exergetic efficiency by taking the ratio of the reversible work to the actual work [21,24]. The reversible work is the difference in exergy flow of states 1 and 6 [21]:

(28) $$w_{rev}=\psi_{6}-\psi_{1}=h_{6}-h_{1}-T_{0}\left(s_{6}-s_{1}\right)$$

The simple Linde-Hampson subsystem exergy efficiency is:

(29) εsLH=wrev(W˙CY)

The pre-cooled Linde-Hampson subsystem exergy efficiency must also account for the from work input to HX-1′:

(30) εpcLH=wrev(W˙CY+W˙HX1′)

### 4.2. Energy Production Subsystem

The ideal pump is assumed to be isentropic, which results in an exergy destruction rate of:

(31) I˙pm˙e=−W˙pm˙e+h6−h7

The exergetic efficiency of the pump is:

(32) εp=ψ7−ψ6(−W˙pm˙e)

Assuming the mass flow rates ${\dot{m}}_{a}$ and ${\dot{m}}_{e}$ are equivalent, the exergy destruction and exergetic efficiency of HX-2 is:

(33) I˙tm˙e=(ψc−ψd)+(ψ7−ψ8)

(34) εHX2=(ψ7−ψ8)(ψd−ψc)

The ideal turbine is assumed to be isothermal, which results in an exergy destruction rate of:

(35) I˙tm˙e=−W˙tm˙e+ψ8−ψ9

The exergetic efficiency of the turbine is:

(36) εt=ψ9−ψ8(−W˙tm˙e)

### 4.3. Complete LAES System

The total exergy destruction rate of the system is the sum of all component exergy destruction rates from Equations (19), (22), (24), (26), (31), (33) and (35). The exergetic efficiency for the entire LAES system is defined as [22]:

(37) εsys=W˙act−I˙totW˙act

where ${\dot{W}}_{act}$ is the sum of all work input:

(38) $${\dot{W}}_{act}={\dot{W}}_{C}+{\dot{W}}_{p}+{\dot{W}}_{HX_{1^{\prime }}}+{\dot{W}}_{HX_{2}}$$

## 5. Results and Discussion

### 5.1. First Law Results and Discussion

The following results use values of enthalpy and entropy gathered from [25]. The tables available from Lemmon et al. provide only discrete iso-pressure values as shown in Table 1.

**Figure 3:**
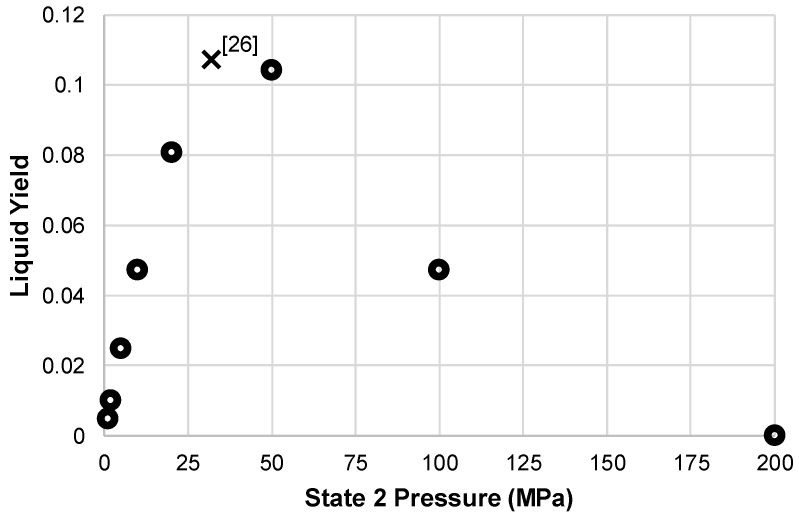
Liquid yield for simple Linde-Hampson subsystem over varying state 2 pressures.

The analysis found that the pressure range for the liquefaction subsystem to produce any liquid yield is 5 MPa to 100 MPa; at 200 MPa, the liquid yield drops to zero. Figure 3 shows the liquid yield for the simple Linde-Hampson subsystem. These results match results found in [9,26]. The maximum yield point found by Joshi and Patel was 0.107 at a pressure of 32 MPa, which is represented on Figure 3 with an ‘x.’

**Figure 4:**
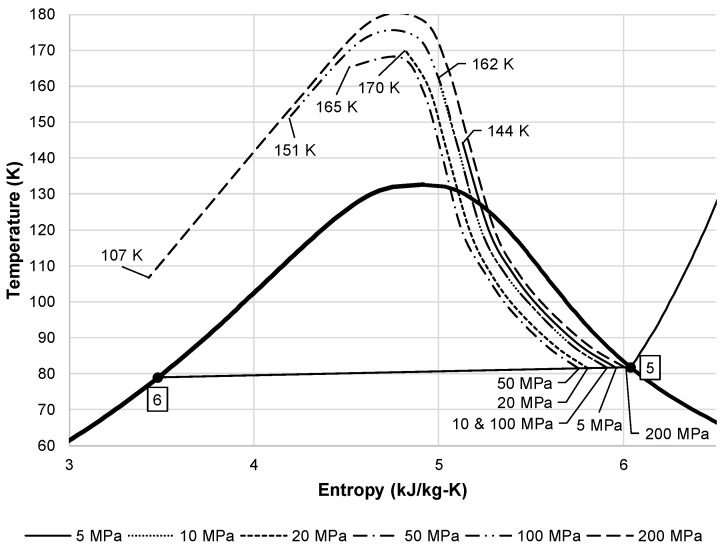
Air temperature entropy diagram showing liquid yield with lines of constant enthalpy from state 3 to state 4.

Figure 4 shows an alternative view of the liquid yield for the simple Linde-Hampson subsystem. As Figure 2 shows, the expansion of air from state 3 to 4 is an isenthalpic process. This throttling process must follow the lines of constant enthalpy shown on a temperature entropy diagram. Figure 4 shows the lines of constant enthalpy followed over the different state 2 pressures. This figure also shows the corresponding state 3 temperature for each state 2 pressure. The state 3 temperature is important when considering the required effectiveness of a heat exchanger to reach this temperature.

**Figure 5:**
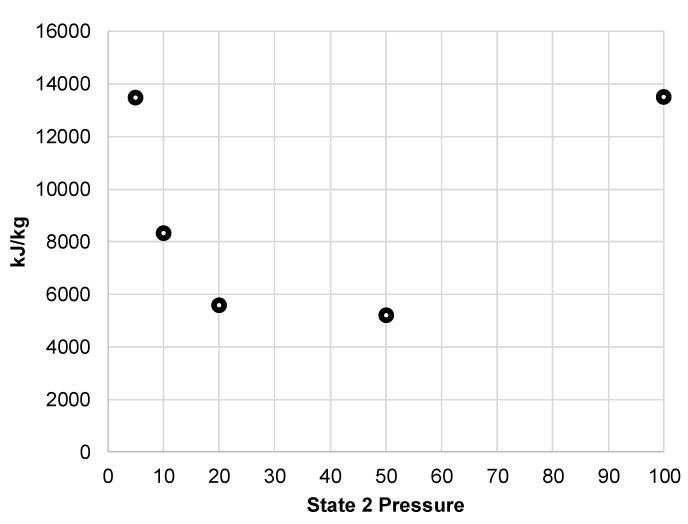
Compressor work per unit mass liquefied for simple Linde-Hampson subsystem.

**Figure 6:**
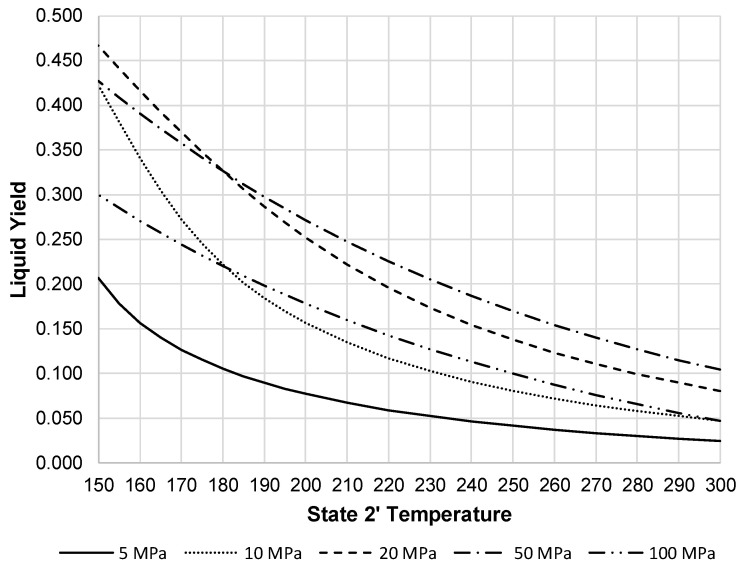
Liquid yield for pre-cooled Linde-Hampson subsystem with varying state 2 pressures.

Displayed in Figure 5, the resulting compressor work per unit mass liquefied shows the optimal state 2 pressure is around 20 to 50 MPa. The liquid yield of this system is likely to be a key measure of performance for a building-scale LAES system. Quickly replenishing liquid air supply ensures continued support during peak demands. Figure 6 shows the liquid yield for the pre-cooled Linde-Hampson subsystem. This figure clearly shows the improvement of liquid yield over all pressures with pressures of 20 and 50 MPa producing the highest liquid yield.

**Figure 7:**
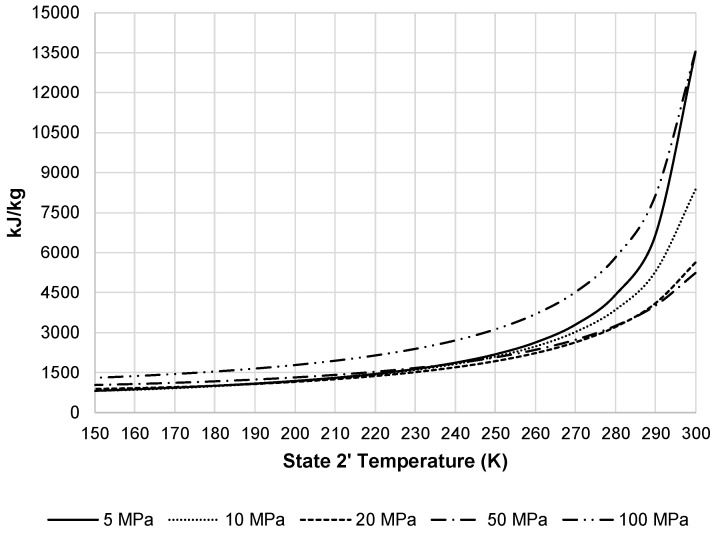
Compressor work for pre-cooled Linde subsystem with varying state 2 pressures.

When the liquid increases, this reduces the work required by the compressor on a per unit mass liquefied basis. Figure 7 shows the resulting compressor work per unit mass liquefied for the pre-cooled Linde-Hampson subsystem. This figure shows all pressures begin to converge, except 100 MPa, at a state 2′ temperature around 250 K.

**Figure 8:**
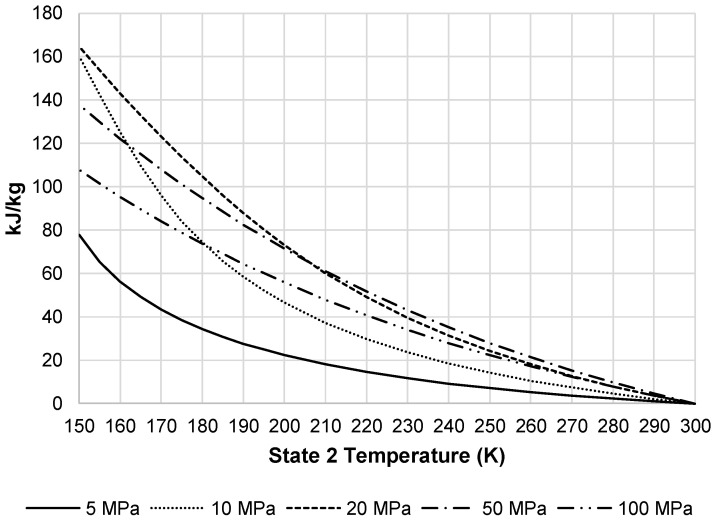
Required heat removal for pre-cooled Linde-Hampson subsystem.

**Figure 9:**
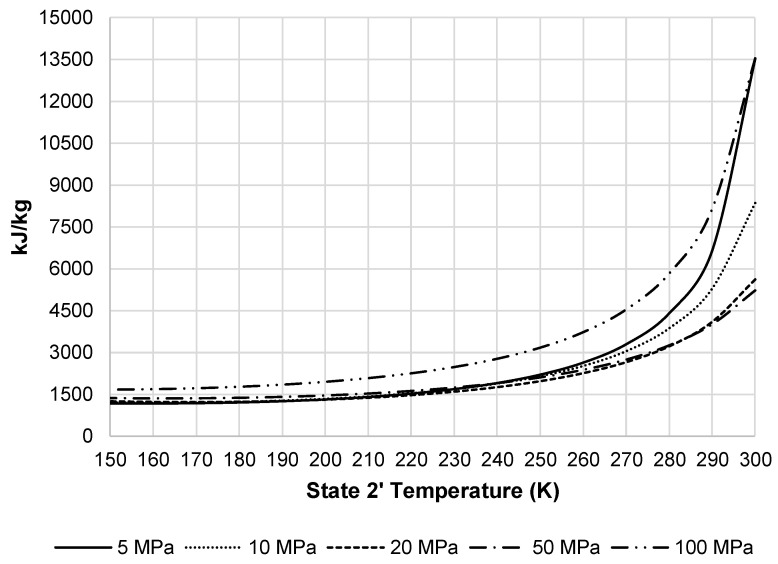
Total work per unit mass liquefied for the pre-cooled liquefaction subsystem.

In order to achieve a desired state 2′ temperature, the system must remove additional heat from the pressurized air stream in HX-1′. Figure 8 displays the additional heat removed from this stream by the external black box system. As the required heat removal increases, required total work of the liquefaction subsystem increases. Figure 9 presents the total work required by the pre-cooled liquefaction subsystem. This figure shows the same convergence of pressures as Figure 7, but also displays the impact HX-1′. The required work at temperatures below 220 K begins to level out and then begins to increase at approximately 150 K.

**Figure 10:**
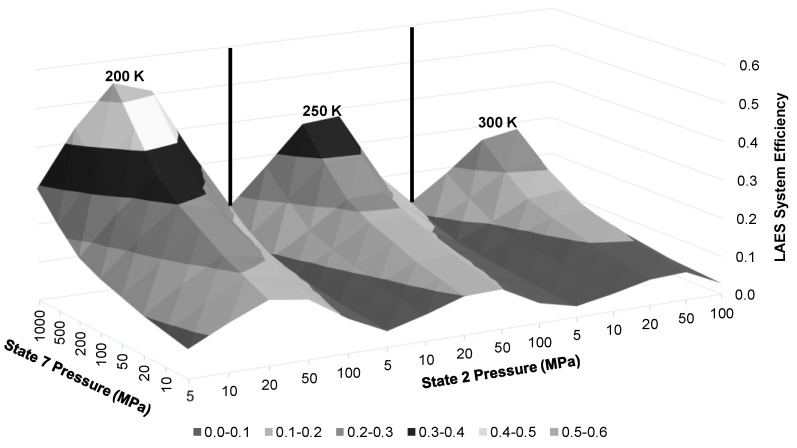
Resulting LAES system energy efficiency with varying state 2 and 7 pressures and three state 2′ temperatures.

The LAES system efficiency is dependent on multiple factors, including state 2 pressure, state 2′ temperature, state 7 pressure, and state 8 temperature. Figure 10 displays the resulting LAES system energy efficiency when using state 2 and 7 pressures and state 2′ temperature as chosen factors. There is a clear increase of system efficiency as the state 2′ temperature is reduced and state 7 pressure is increased. Similar to the compressor work per unit mass liquefied results in Figure 7, the LAES system efficiency peaks at state 2 pressures around 20 and 50 MPa.

**Figure 11:**
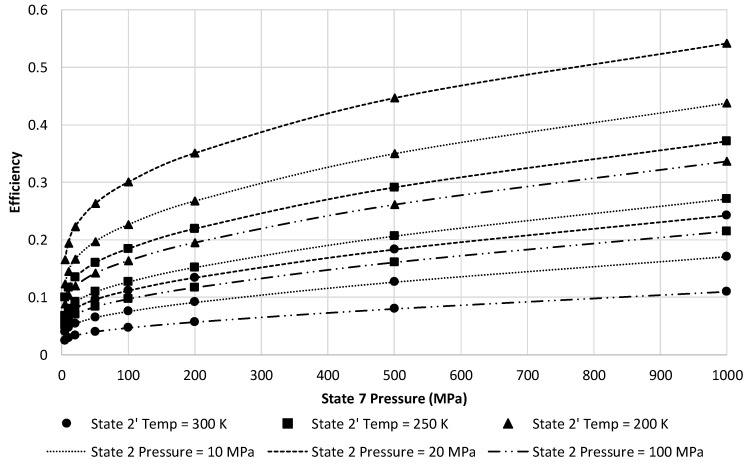
Resulting LAES system energy efficiency over full range state 7 pressures and three selected State 2′ temperatures and state 2 pressures.

Figure 11 presents a narrower view of Figure 10 by reducing to only three state 2 pressures of 10, 20, and 100 MPa. This figure depicts a clear gain in energy efficiency choosing 20 MPa and lower state 2′ temperatures. Although the ranges presented on the figure may not be achievable in a building-scale LAES system. Maximum state 7 pressure is likely to be approximately 100 MPa given size restrictions for a building-scale system. A state 2′ temperature of 250 K is likely to be attainable. 

**Figure 12:**
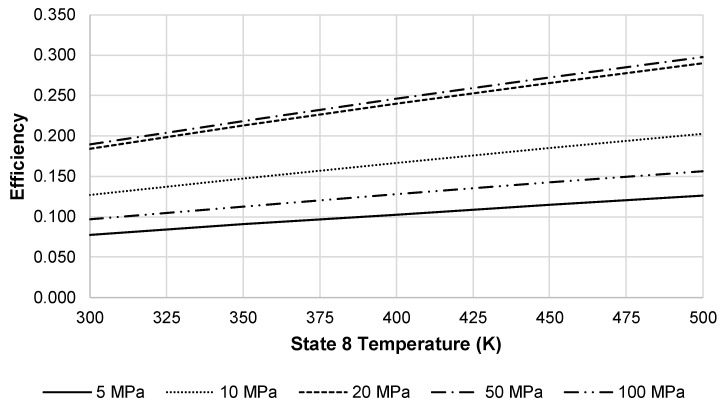
Effects of increasing state 8 temperature on LAES energy efficiency at a state 2′ temperature of 250 K and state 7 pressure of 100 MPa.

At these factors, the approximate LAES system efficiency at 20 MPa is 18.4%. Increasing the temperature into the turbine inlet increases the energy efficiency of the LAES system. Figure 12 depicts this increase in energy efficiency using a state 2′ temperature of 250 K and state 7 pressure of 100 MPa. Although not depicted in Figure 12, there is a maximum temperature where gains in efficiency would reach an asymptotic limit.

### 5.2. Second Law Results and Discussion

The exergy destruction and exergetic efficiency of the compressor, HX-1′, the pump, HX-2, and the turbine are trivial due to using ideal cases. The exergy destruction for each of these components are zero and the exergetic efficiency is equal to one. Although this is not the case for the turbine when the temperature at state 8 exceeds 300 K. The largest attributer to exergy destruction in this system is the JT valve.

**Figure 13:**
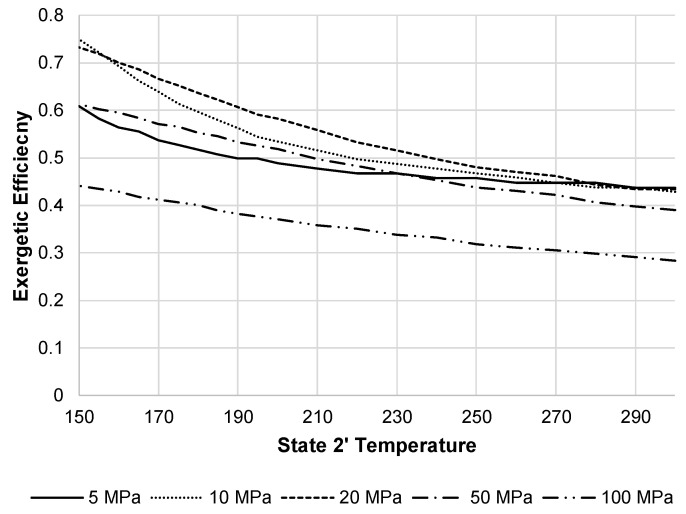
JT valve exergetic efficiency with varying state 2 pressures and state 2′ temperatures.

Figure 13 shows the JT valve exergetic efficiency over different state 2 pressures when decreasing the state 2′ temperature. The optimal state 2 pressure in terms of exergetic efficiency for the JT valve is 20 MPa when state 2′ temperatures are below 270 K.

**Figure 14:**
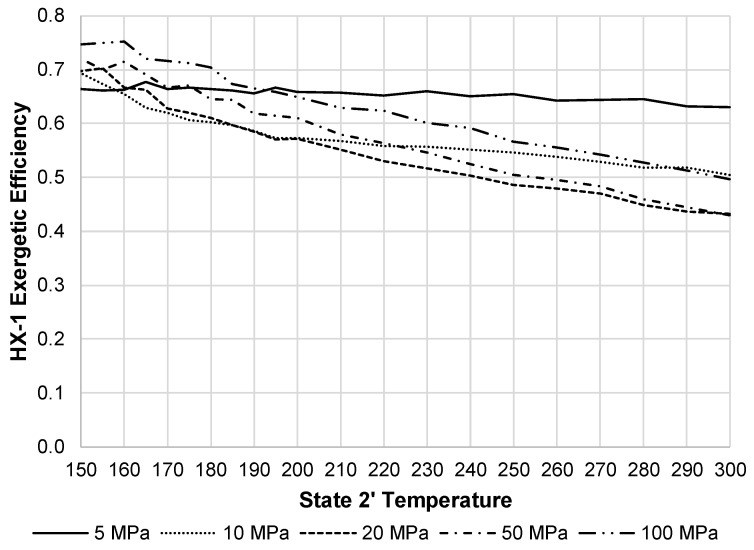
HX-1 exergetic efficiency with varying state 2 pressures and state 2′ temperatures.

**Figure 15:**
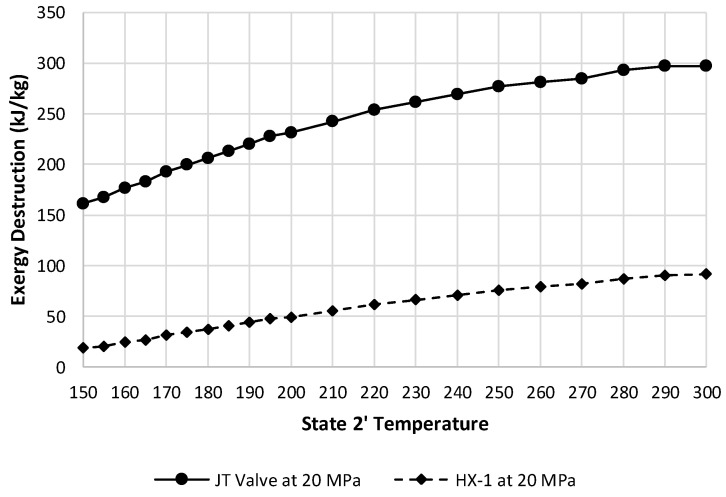
Exergy destruction comparison of the JT valve and HX-1 at a state 2 pressure of 20 MPa over varying state 2′ temperatures.

Figure 14 displays the exergetic efficiency of HX-1 over varying state 2′ temperatures and state 2 pressures. This component shows 20 MPa as the worst pressure in terms of exergetic efficiency. Although, at this pressure, the exergy destruction rate of HX-1 is substantially less than the JT valve, and therefore, has less impact on the overall system exergetic efficiency. Figure 15 depicts these two components exergy destructions at a state 2 pressure of 20 MPa.

**Figure 16:**
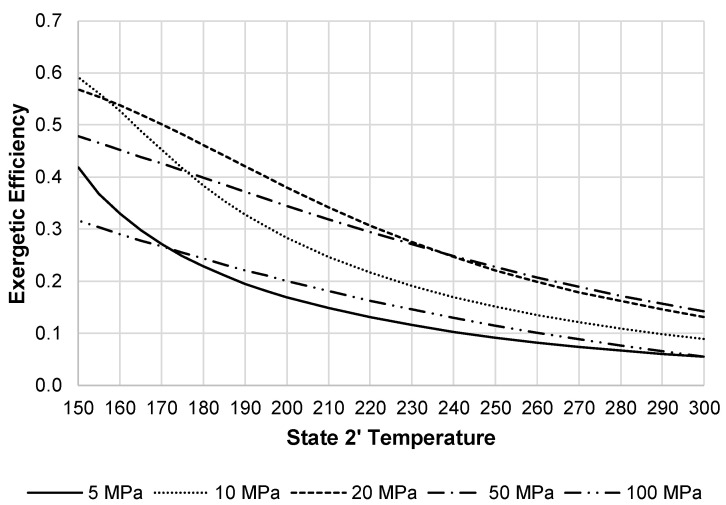
Exergetic efficiency of the liquefaction subsystem.

**Figure 17:**
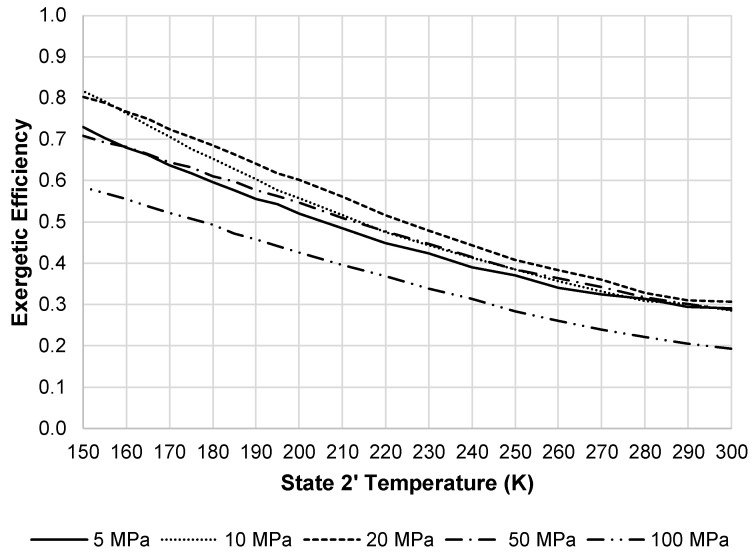
LAES system exergetic efficiency at a state 7 pressure of 100 MPa and state 8 temperature of 300 K.

Figure 16 shows the exergetic efficiency of the liquefaction subsystem. This figure again shows that 20 MPa to 50 MPa are an optimum pressure range. Figure 17 displays the changes in the LAES system exergetic efficiency when changing the state 2′ temperature and pressure. The optimum pressure displayed on the figure is 20 MPa. 

## 6. Conclusions

This paper conducted an energy and exergy analysis of an ideal, combined liquid air energy storage and expansion system. The results showed that the pre-cooled Linde-Hampson subsystem was superior to the simple Linde-Hampson subsystem and heating liquid air beyond ambient temperature was superior to heating to only ambient. The optimal state 2 pressure range was 20 to 50 MPa where there was maximum liquid yield. Pre-cooling resulted in an increase in liquid yield, energy efficiency, and exergy efficiency. Although, pre-cooling below 150 K will result in increased total work per unit mass liquefied. Heating the liquid air beyond ambient temperature results in increased electrical generation and increases the overall efficiency of the system.

Additional improvement of the energy and exergy efficiency may be found with alternative liquefaction subsystems, which utilize an expander, such as the Claude and Heylandt systems. The system would be improved further by incorporating cold and heat recovery systems, as used by previous work which achieved round trip efficiencies of 45–57%.

## Figures and Tables

**Table 1:** List of iso-pressures available from Lemmon et al. [25].

**Pressure (MPa)**	0.101325	0.2	0.5	1	2	5	10	20	50	100	200	500	1000	2000

## References

[1] Luo X., Wang J., Dooner M., Clarke J. (2015). Overview of Current Development in Electrical Energy Storage Technologies and the Application Potential in Power System Operation. Appl. Energy.

[2] Chen H., Cong T.N., Yang W., Tan C., Li Y., Ding Y. (2009). Progress in Electrical Energy Storage System: A Critical Review. Prog. Nat. Sci..

[3] Wang S., Xue X., Zhang X., Guo J., Zhou Y., Wang J. (2015). The Application of Cryogens in Liquid Fluid Energy Storage Systems. Phys. Proc..

[4] Krawczyk P., Szablowski L., Karellas S., Kakars E. (2018). Comparative Thermodynamic Analysis of Compressed Air and Liquid Air Energy Storage Systems. Energy.

[5] McLarnon F.R., Cairns E.J. (1989). Energy Storage. Annu. Rev. Energy.

[6] Kim Y.M., Lee J.H., Kim S.J., Favrat D. (2012). Potential and Evolution of Compressed Air Energy Storage: Energy and Exergy Analyses. Entropy.

[7] Kim Y., Favrat D. (2010). Energy and Exergy Analysis of a Micro-Compressed Air Energy Storage and Air Cycle Heating and Cooling System. Energy.

[8] Akhurst M., Arbon I., Ayres M., Brandon N., Bruges R., Cooper S., Ding Y., Evison T., Goode N., Grünewald P. (2013). Liquid Air in the Energy and Transport Systems Opportunities for Industry and Innovation in the UK.

[9] Barron R.F. (1985). Cryogenic Systems.

[10] Lim Y., Al-Atabi M., Williams R.A. (2016). Liquid Air as an Energy Storage: A Review. J. Eng. Sci. Tech..

[11] Smith E. (1977). Storage of Electrical Energy Using Supercritical Liquid Air. Proc. Inst. Mech. Eng..

[12] Giuizzi G.L., Manno M., Tolomei L.M., Vitali R.M. (2015). Thermodynamic Analysis of a Liquid Air Energy Storage System. Energy.

[13] Kawczyk P., Szablowski L., Badyda K., Karellas S., Kakaras E. (2016). Impact of Selected Parameters on Performance of the Adiabatic Liquid Air Energy Storage System. J. Power Technol..

[14] Xue X., Wang S., Zhang X., Cui C., Chen L., Zhou Y., Wang J. (2015). Thermodynamic Analysis of a Novel Liquid Air Energy Storage System. Phys. Proc..

[15] Abdo R.F., Pedro H.T., Koury R.N., Machado L., Coimbra C.F., Porto M.P. (2015). Performance Evaluation of Various Cryogenic Energy Storage Systems. Energy.

[16] Yu J., Tian G., Xu Z. (2009). Exergy Analysis of a Joule-Thomson Cryogenic Refrigeration Cycle with an Ejector. Energy.

[17] Moran M.J., Shapiro H.N. (2004). Fundamentals of Engineering Thermodynamics.

[18] Rosen M.A., Dincer I. (2001). Exergy as the Confluence of Energy, Environment, and Sustainable Development. Exergy Int. J..

[19] Lior N., Zhang N. (2007). Energy, Exergy, and Second Law Performance Criteria. Energy.

[20] Kotas T.J. (1985). The Exergy Method of Thermal Plant Analysis.

[21] Kanoglu M., Dincer I., Rosen M.A. (2008). Performance Analysis of Gas Liquefaction Cycles. Int. J. Energy Res..

[22] Kanoglu M. (2002). Exergy Analysis of Multistage Cascade Refrigeration Cycle Used for Natural Gas Liquefaction. Int. J. Energy Res..

[23] Paniagua I.L., Martin J.R., Fernandez C.G., Alvaro A.J., Carlier R.N. (2013). A New Simple Method for Estimate Exergy Destruction in Heat Exchangers. Entropy.

[24] Borri E., Tafone A., Romagnoli A., Comodi G. (2017). A Preliminary Study on the Optimal Configuration and Operating Range of a "Microgrid Scale" Air Liquefaction Plant for Liquid Air Energy Storage. Energy Conserv. Manag..

[25] Lemmon E.W., Jacobsen R.T., Penoncello S.G., Friend D.G. (2000). Thermodynamic Properties of Air and Mixtures of Nitrogen, Argon, and Oxygen From 60 to 2000 K at Pressures to 2000 MPa. J. Phys. Chem. Ref. Data.

[26] Joshi D., Patel H. (2015). Analysis of Cryogenic Cycle with Process Modeling Tool: Aspen HYSYS. J. Instrum..

